# Codon Pattern and Compositional Constraints Determination of Genes Associated with Chronic Periodontitis

**DOI:** 10.3390/genes13111934

**Published:** 2022-10-24

**Authors:** Rekha Khandia, Megha Pandey, Igor Vladimirovich Rzhepakovsky, Azmat Ali Khan, Isabel Legaz

**Affiliations:** 1 Department of Biochemistry and Genetics, Barkatullah Universty, Bhopal 462026, India; 2 Translational Medicine Center, All India Institute of Medical Sciences, Bhopal 462020, India; 3 Medical and Biological Faculty, North Caucasus Federal University, 355009 Stavropol, Russia; 4 Pharmaceutical Biotechnology Laboratory, Department of Pharmaceutical Chemistry, College of Pharmacy, King Saud University, Riyadh 11451, Saudi Arabia; 5 Department of Legal and Forensic Medicine, Biomedical Research Institute (IMIB), Regional Campus of International Excellence “Campus Mare Nostrum”, Faculty of Medicine, University of Murcia, E-30120 Murcia, Spain

**Keywords:** chronic periodontitis, inflammatory disease, codon usage, genetics relationship of periodontitis

## Abstract

Genome-wide association studies showed the relationship of *NIN*, *ABHD12B*, *WHAMM*, *AP3B2*, and *SIGLEC5* with chronic periodontitis. The study’s objective was to investigate different molecular patterns and evolutionary forces acting on the mentioned genes. The investigation of molecular patterns encompasses the study of compositional parameters, expression profile, physical properties of genes, codon preferences, degree of codon bias, determination of the most influential codons, and assessment of actions of evolutionary forces, such as mutations and natural selection. The overall compositional analysis revealed the dominance of A and G nucleotides compared to T and C. A relatively low codon usage bias is observed. The CTG codon is the most overused codon, followed by TCC. The genes, *AP3B2* and *SIGLEC5*, preferred GC-ending codons, while *NIN*, *ABHD12B*, and *WHAMM* preferred AT-ending codons. The presence of directional mutational force and natural selection was found to operate codon usage in genes envisaged, and selective forces were dominant over mutational forces. Apart from mutation and selection forces, compositional constraints also played imperative roles. The study enriched our knowledge of specific molecular patterns associated with the set of genes significantly associated with chronic periodontitis. Further studies are warranted to identify more genetic signatures associated with the disease.

## 1. Introduction

A study on the global burden of diseases, injuries, and risk factors [1] demonstrated that dental caries in permanent teeth and periodontitis were the primary and 11th most prevalent reasons for ailments worldwide. The etiology of this disease needs to be characterized as it confers both health and financial burdens [2,3]. Genetic factors contribute to oral health and the heritability of dental caries to an extent as high as 50% [4]. Characterizing these genetic factors will help us understand the disease and develop countermeasures to fight these diseases. Chronic periodontitis (CP) negatively influences glycemic control, and diabetes coupled with CP enhances the risk of cardiorenal mortality [5]. The CP patients demonstrate significant endothelial dysfunction [6], and studies revealed a close association between periodontitis and cardiovascular diseases [7], liver diseases [8], rheumatoid arthritis [9], alopecia areata [10], anxiety-like behaviors [11], respiratory diseases [12], and neuropathy in diabetic patients [13]. In addition, studies have shown that CP enhances the risk of preeclampsia in pregnant women [14]. Chronic periodontitis (CP) involves host inflammatory/immune response against the subgingival biofilm [15]. A study encompassing a total of 3160 study subjects revealed that the *NIN* and *SIGLEC5* genes are associated with CP susceptibility [16]. Gene-centric association analysis based on the Gene Ontology, Ingenuity, KEGG, Panther, Reactome, and Biocarta databases for gene set enrichment analyses that included the study of 18,307 genes; 4 genes (*NIN*, *ABHD12B*, *WHAMM*, and *AP3B2*) showed significant association with CP [17]. 

Amino acids are the protein molecule’s basic unit, and a codon encodes for an amino acid. Except for methionine and tryptophan, all the amino acids are encoded by two or more codons, and such codons are called synonymous codons. In any organism, all the synonymous codons are not used equally, and there is unequal usage of a codon in any gene, tissue, or organism and is called codon usage bias. Codon bias can be gene-specific [18], species-specific [19], tissue-specific [20,21], or function-specific [22,23]. Codon bias is often determined by compositional, mutational, or selectional forces [24]. Codon bias plays a crucial determinant of gene expression levels mainly via influencing transcription [25], and reports suggest more substantial bias in the case of highly expressed genes due to selection pressure [26].

Periodontitis affects 11% of the world’s population and poses a tremendous public health challenge [27]. Studies have been conducted on the association of CP with various disorders; however, no work has been reported on codon usage studies in genes related to CP. The present study encompasses the study of 30 transcripts belonging to five genes, *NIN*, *ABHD12B*, *WHAMM*, *AP3B2*, and *SIGLEC5*. The function of the *NIN* gene includes correct positioning and anchoring of microtubule minus ends. *ABHD12B* regulates immune and neurological processes, *WHAMM* participates in Arp2/3-mediated actin polymerization, *AP3B2* is involved in neurotransmitter release, and *SIGLEC5* performs sialic-acid dependent cell binding. Since the underlying molecular mechanisms of periodontitis are still unclear, the effects of the *NIN*, *ABHD12B*, *WHAMM*, *AP3B2*, and *SIGLEC5* genes on periodontitis have not been elucidated [16].

The study provides insight into various factors influencing codon bias. The study also elucidates the over- or under-represented codons and the codons having maximum influence in codon usage. Additionally, the study will help identify molecular signatures associated with the CP-associated genes and design synthetic gene constructs for gene therapy purposes to manipulate gene expression levels, where an aberrant gene expression level is responsible for ailments.

## 2. Materials and Methods

### 2.1. Sequence Retrieval

In this study, the coding transcripts of the genes (S1) associated with CP were retrieved from the National Center for Biotechnology Information (NCBI) GenBank database (http://www.ncbi.nlm.nih.gov accessed on 20 March 2022). Upon investigating the literature, we found only five genes responsible for CP (*NIN*, *ABHD12B*, *WHAMM*, *AP3B2*, and *SIGLEC5*). Therefore, we considered all the protein-encoding transcripts for the mentioned genes. The criteria set for the transcripts were that they must be initiated with a start codon and ended with a stop codon and should be devoid of bases other than A, T, C, and G. There were eight, seven, six, five, and four numbers of transcripts that fulfilled the criteria set *for NIN*, *ABHD12B*, *WHAMM*, *AP3B2*, and *SIGLEC5* genes, respectively.

### 2.2. Compositional Analysis

The overall nucleotide composition of the transcripts was determined. The nucleotide composition at the first and third codon positions (%A, %T, %C, %G, %A3, %T3, %C3, %G3) were determined. Overall %AT and %AT3 and %GC and %GC3 were also determined. Analyses of %GC3 (GC composition at the third codon position) and %GC12 (composition at the first and second codon) were also done. %GC3 helps evaluate the compositional and codon bias [28].

### 2.3. Relative Synonymous Codon Usages (RSCU)

RSCU is the ratio of the observed frequency of codons to the expected frequency of the codon [29]. All transcripts’ RSCU values were calculated using the CAIcal server [30]. RSCU values above 1.6 and below 0.6 were considered overrepresented and underrepresented, respectively. The values between 0.6 and 1.6 are taken as randomly used, and codons are called unbiased [31].

### 2.4. Codon Adaptation Index (CAI)

CAI is a measure to estimate protein expression levels and determine the effect of natural selection that helps shape the codon usage pattern in transcripts [32]. If all the synonymous codons are used equally, the CAI value will be 0, while in the case of the strongest bias, the value of 1 will be obtained [33]. Higher CAI values show both high expression as well as high bias. CAI was calculated using the formula given by Sharp and Li (1987) [31].

### 2.5. Effective Number of Codons (ENc)

ENc is a commonly used indicator of codon usage bias [34]. The values of ENc range between 20–61. It is a non-directional measure of codon bias, and the value 20 shows the highest bias, while 61 shows no bias. The value 20 indicates that only one codon will be used for a single amino acid, while the value 61 indicates that all the synonymous codons are equally used. Generally, genes with ENc values below 35 are considered highly biased, while ENc above 50 suggests low bias [35,36].

### 2.6. PR2-Bias Plot Analysis

The intra-strand base composition theoretically should be A = T and G = C which is called the type 2 parity rule or PR2, and violation of this rule is expected in amino acids encoded by four codons [37]. It is plotted with AT-bias value [A3/(A3+ T3)] as the ordinate and GC-bias [G3/(G3 + C3)] as the abscissa. In this plot, the center is 0.5, which is the place where A = T and G = C (PR2), indicating no bias. The center shows no bias between the two strands of DNA for mutation and selection rates [36]. A value less than 0.5 indicates a preference for pyrimidine over purine [38].

### 2.7. Neutrality Plot

Regression analysis between %GC3 and %GC12 (mean %GC1 and %GC2) is a standard method to explore the effects of natural selection and mutation pressure [39]. It determines which evolutionary force majorly influences codon usage [40]. %GC3 and %GC12 are plotted on lateral and vertical axes to produce a scatter plot serving as the neutrality plot. When the slope value is 1, it is suggestive of codon usage bias solely driven by neutrality. The effects of other forces are determined by its deviation from neutrality [41].

### 2.8. Principal Component Analysis (PCA)

PCA is a commonly employed multivariate statistical technique to analyze the trends in codon usage patterns. Here, we used PCA to obtain significant trends in the transcripts of genes associated with enhanced risk of CP. The method reduces the original variables to a lower number of principal components without losing its information. Each transcript is represented as a 59-dimensional vector (the number of possible codons excluding stop codons, methionine, and tryptophan encoded by single codons). Finally, we took the top two principal components with the most significant variance. We performed PCA analysis using Origin18 software (https://www.originlab.com/demodownload.aspx, accessed on 19 September 2022).

### 2.9. Phylogenetic Analysis

To explain any phylogenetic relationship between the gene transcripts, phylogenetic analysis was conducted using the hierarchical clustering method and Ward’s algorithm based on RSCU values with 1000 bootstrap values. PAST4 [42] and Molecular Evolutionary Genetics Analysis 10 (MEGA-X) (version 10.1.8) were used for the study. 

## 3. Results

### 3.1. Nucleotide Composition Affects the Codon Usage Bias

**Figure 1:**
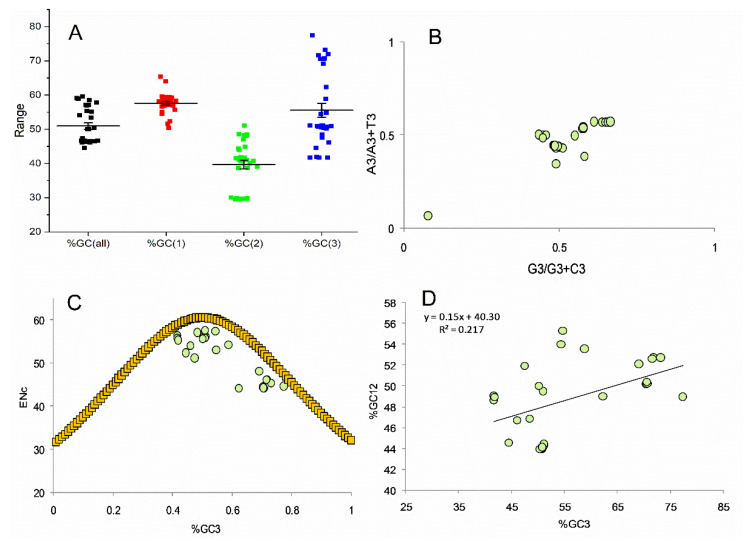
(**A**). Overall %GC composition and %GC composition at all the three codon positions (**B**). Parity pot analysis (**C**). ENc-GC3 analysis (**D**). Neutrality plot.

Reports suggest the impact of compositional constraints on codon bias [43]; therefore, overall nucleotide composition and AT and GC composition at various codon positions were determined for all the 30 transcripts envisaged. Compositional features of the transcripts reveal the dominance of nucleotide A. Average nucleotide composition analysis revealed that percent composition A was highest (27.81 ± 4.67), followed by nucleotide G (26.93 ± 2.17). Percent composition for nucleotide T was least (21.24 ± 2.42). Percent composition at the third codon position was highest for nucleotide G (30.18 ± 4.91) followed by C (25.38 ± 8.6). The results indicate that though the average composition of A nucleotide is highest, the composition of nucleotide G was highest at the third codon position. On the other hand, the percent composition was least for nucleotide T, both overall and at the third codon position. The percent composition of the %A3 and %T3 were almost similar (22.32 ± 6.96 and 22.1 ± 5.39 for A3 and T3, respectively). The GC and AT compositions were nearly equal (50.95 ± 5.24 and 49.0 ± 5.24 for GC and AT compositions, respectively). The results indicated that both the GC and AT contents are used equally in the transcripts. However, the GC and AT compositions at the third codon positions differed, and %GC3 (55.57 ± 11.07) was higher than %AT (44.42 ± 11.07). When the %GC compositions at all three codon positions were evaluated, it was observed that %GC3 composition is the most variable, while %GC2 composition is the least (Figure 1A).

### 3.2. RSCU Analysis

In most sequenced genomes, synonymous codons are not used in equal frequencies. This phenomenon is termed codon-usage bias. RSCU is a measure of codon bias [44]. Codons with RSCU values >1.6 and <0.6 are called overrepresented and underrepresented, respectively, while values between 0.6 and 1.6 are called randomly used codons [45]. The analysis revealed that codon CTG varied largely, and its RSCU values ranged between 1.03 (random usage) and 5.16 (extremely biased usage). In contrast, codon ACG varied the least with RSCU values ranging between 0 and 0.69. Out of six CG-containing codons, TCG, CCG, ACG, GCG, CGA, and CGT, the codons ending with CG (TCG, CCG, ACG, GCG) had more bias than all CG-containing codons and were underrepresented in more than 75% of genes envisaged. Out of six AT-containing codons, only one codon, CTA, was underrepresented in more than 75% of genes. The RSCU values of envisaged genes are given in Table 1. 

CAI is a geometric mean of the RSCU values of codons of genes and is measured using a trained codon usage table [46]. ENc is also a non-directional measure of codon usage bias [36]. CAI and ENc values were correlated with overall %GC and %GC composition at all three codon positions. The correlation analysis revealed that CAI had a significant negative correlation with ENc, which indicated that with the increasing bias, gene expression also increased in the case of transcripts associated with CP (since ENc is a non-directional measure of codon bias). Furthermore, CAI showed a positive correlation with %GC, %GC2, and %GC3, while ENc showed a negative correlation (Table 2). The results are indicative of the effects of compositional constraints on codon usage.

### 3.3. At the Third Codon Position, G and T Are Preferred

Parity analysis gives information regarding the skew of A/T and C/G nucleotides at the third codon position. In the presence of only mutational force, CG and AT will be equally used, while unequal usage is not entirely explained by natural selection [47]. The average GC bias (%G3/%G3 + %C3) was 0.551 ± 0.07, and AT (%A3/%A3 + %T3) bias was 0.495 ± 0.065. The average value indicated that G is preferred over C and T over A (Figure 1B).

### 3.4. Selection Is Dominant Force Affecting Codon Usage Evidenced by ENc-GC3 Plot

A significant positive correlation has been observed between the %GC12 and %GC3 contents (r = 0.466, *p* < 0.01), which indicates that the directional mutational forces are working on all GC contents at all three codon positions [36].The ENc-GC3 plot is constructed to assess compositional, selectional, and mutational forces acting on CP-related genes. The expected Nc (ENc) is presented as a curve in Figure 1C [35]. The GC3 content ranged between 41.70% and 77.46%, and the ENc value ranged between 44.05 and 57.51, pointing toward the low codon bias. The ENc-GC3 analysis is considered an alternative to the multivariate statistical analysis method to reveal synonymous codon usage and determine the forces involved in shaping codon usage. The ENc line suggests composition-biased mutation pressure on codon usage, and selection pressure is zero if all the data points are present over the expected line [48]. In the case of translational selection, the data point will lie too far from the curve [49]. In the present study, the role of selection pressure appears to be significant (Figure 1C).

### 3.5. Selection Is Dominant Force Affecting Codon Usage Evidenced by Neutrality Plot

A neutrality plot was constructed between %GC3 and %GC12. %GC12 is the average value of %GC composition at the first and second codon positions. The neutrality plot (Figure 1D) shows the equilibrium between mutation and selection. The equilibrium near zero shows high neutrality or mutation forces, while values near one show high selection forces [50]. The model depicted that 21.77% of the variation in %GC12 results from GC3 component variation [26].

The neutrality plot reveals the impact on mutational and selection forces on codon usage [41]. To determine what major force is driving codon usage bias in envisaged genes, *NIN*, *ABHD12B*, *WHAMM*, *AP3B2*, and *SIGLEC5*, the neutrality plot was generated. The slope of the estimated equation revealed that mutation pressure contributed a minor role of 15%, while selection (85%) played the main role in determining codon usage (Figure 1D).

### 3.6. Principal Component Analysis (PCA) Revealed the Effect of Composition, Gene Expression, and Protein Properties on Codon Usage

**Figure 2:**
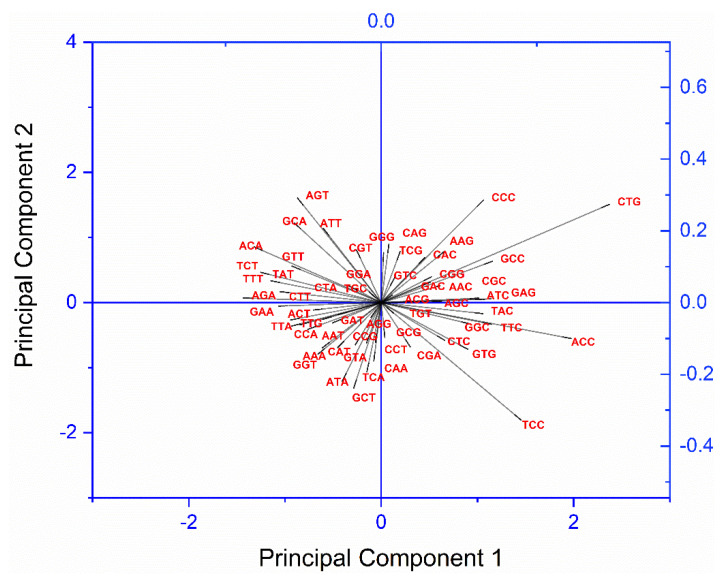
PCA biplot analysis revealed that CTG codon is maximally influencing the overall codon usage.

PCA is a method to reduce the number of variables without compromising the information. The PCA done for 30 transcripts revealed that the first two PCs contributed 49.93% and 14.80% variation, respectively. PCA biplot analysis reveals the effect of each codon based on its loading values. The length of the arrow is proportional to its contribution to variation. Therefore, the lengthiest the arrow, the more contribution towards variation [51]. In the present study, out of 59 codons, codon CTG contributed to maximum variation, followed by TCC (Figure 2). GC-ending codons were preferred by *AP3B2* and *SIGLEC5* gene transcripts, while AT-ending codons were preferred by *NIN*, *ABHD12B*, and *WHAMM.*

Furthermore, we performed correlation analysis of PC1 and PC2 with other variables (Table 3). The analysis revealed that both PC1 and PC2 are correlated with few of the compositional constraints, suggestive of the impact of composition on codon usage pattern. CAI values ranged between 0.706 and 0.839 with an average of 0.751 ± 0.04, suggestive of good expression level and existence of codon bias [30]. PC1 showed significant correlation with CAI, and PC2 showed significant correlation with protein properties, and it indicated the effect of codon usage bias on both the protein expression and protein properties.

### 3.7. Phylogenetic Analysis

**Figure 3:**
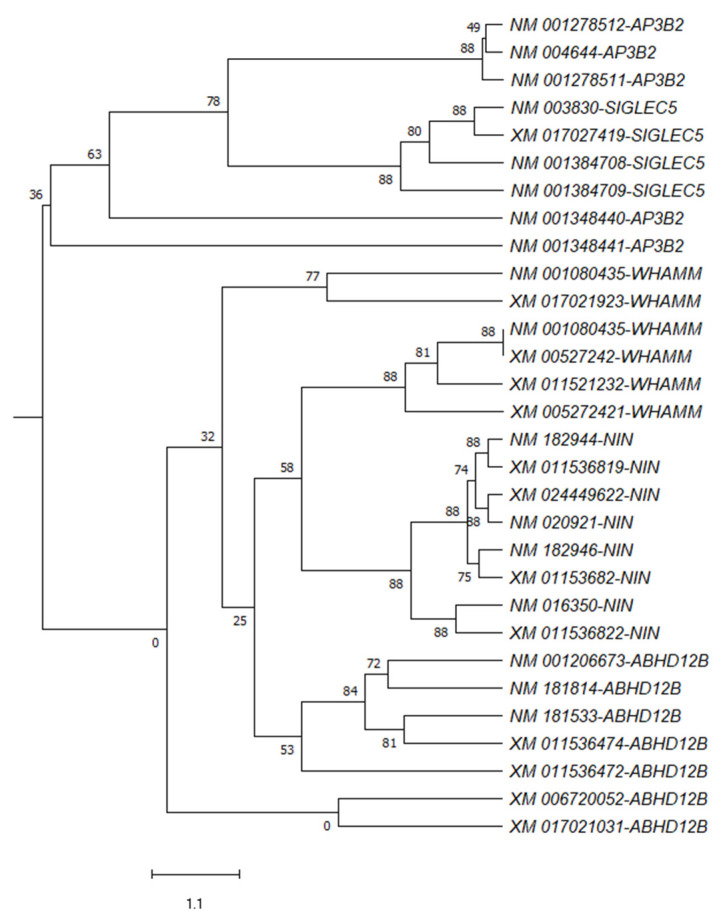
Classical clustering showed the transcripts might be grouped into two clusters. AP3B2 and SIGLEC5 transcripts clustered together, while the rest of the transcripts clustered separately.

Phylogenetic analysis and the evolutionary relationship of 30 transcripts were carried out using MEGA 10 software. Phylogenetic analysis revealed that the genes could be separated mainly into two clusters. One cluster is comprised of *SIGLEC5* and *AP3B2*, while the second cluster is made up of *NIN*, *ABHD12B*, and *WHAMM* transcripts. In the second cluster, *ABHD12B* transcripts were separated from others showing distinct codon usage by these transcripts. From the tree, we can derive that each gene is phylogenetically distinct, and transcripts from a single gene clustered together suggest no common origin (Figure 3).

## 4. Discussion

Periodontitis is a condition of gum infection and inflammation that may result in tooth decay and other serious health complications, including heart problems [52], lung diseases [12], and diabetes [5]. Periodontitis is a chronic inflammatory disorder of the tooth-supporting tissue, and age, race, obesity, and smoking status are a few causative factors. Genome-wide association studies conducted by [16,17] revealed the association of *NIN*, *ABHD12B*, *WHAMM*, *AP3B2*, and *SIGLEC5* with CP. Therefore, we are tempted to undertake molecular studies related to these genes. We performed various studies that revealed molecular signatures and different forces acting on these genes during evolution.

The present study’s average ENc value was 52.35 ± 5.01, indicating a comparatively lower codon usage bias in the envisaged genes. In other diseases associated with human disorders, such as Alzheimer’s, ENc was 48.84 [52], and in Krabbe disease, a rare neurodegenerative disorder, ENc was 56.98 [22]. The results from other studies support our result, which shows that low codon bias might facilitate efficacious replication in vertebrates [43]. The GC content influences the codon usage and modulates the thermostability and bendability of DNA, consequently influencing the transcription process by keeping the DNA region transcribed in unwound status [53]. The genes associated with CP had higher A and G nucleotides than T and C in the present study. The GC component was highest at the third codon position while lowest at the second codon position. Our results concord with Uddin who found in genes associated with anxiety in humans [47]. Furthermore, a significant positive correlation between GC12 and GC3 content (r = 0.466, *p* < 0.01) is suggestive of the action of directional mutation force at all three codon positions and is in concordance with the earlier studies [54]. 

RSCU analyses reveal the overrepresented, underrepresented, and randomly used codons in any given gene. Our analysis revealed that codon CTG is the most overrepresented codon in most transcripts. CTG codon is overrepresented in genes common to immunodeficiency and cancer [23]. Higher CTG representation might be understood based on exaggerated T→C transition rates, resulting in more CTG codons [55]. Out of six CG-containing codons, four CG-ending codons, i.e., TCG, CCG, ACG, GCG, were underrepresented in more than 75% of the gene. The results appear to be a consequence of the CpG dinucleotide depletion in most coding sequences. CpG depletion results from selective forces, including the involvement of CpG in gene silencing and X chromosome inactivation and spontaneous methylation of CpG dinucleotide into TpG [35]. Thus, over-representation of the CTG codon might be considered a result of selective forces. The CTG codon also influenced the overall codon usage the most. A significant correlation of CAI and protein properties (GRAVY and AROMA) with the first two PCs indicated that protein expression and physical properties are affected by changes in codon usage. Cluster analysis is a method where genes with common attributes are clustered together. All the tested gene transcripts were separated into two clusters; one contained *SIGLEC5* and *AP3B2* and *NIN*, *ABHD12B*, and *WHAMM* transcripts. It indicated that the codon usage pattern was similar for the *SIGLEC5* and *AP3B2* gene transcripts, and a slightly different pattern was followed by the *NIN*, *ABHD12B*, and *WHAMM* gene transcripts.

CP negatively influences glycemic control. Diabetes coupled with CP increases cardiorenal mortality risk [5]. The CP patients demonstrated significant endothelial dysfunction [56], and epidemiological studies revealed a close association between periodontitis and cardiovascular diseases [7], liver diseases [8], rheumatoid arthritis [57], alopecia areata [10], anxiety-like behaviors [11], respiratory diseases [12], and neuropathy in diabetic patients [58]. Our study provides information regarding molecular signatures, gene expression patterns, protein properties, codon usage, codon bias, information regarding codons influencing codon usage to various extents, and dinucleotide pattern. The information is also helpful in modulating the gene expression of the envisaged gene. Envisaged genes, *SIGLEC5*, *AP3B2*, *NIN*, *ABHD12B*, and *WHAMM*, in our study are associated with the CP, and gene product quantities influence the pathobiology. We have observed in our work that both the protein expression and physical properties are affected by changes in codon usage; therefore, altering codon usage may also manipulate the gene expression pattern. A synthetic biology approach where a gene can be recoded to obtain the desired gene expression level might be helpful. Using the technology, both the upregulation and downregulation of gene expression are possible. The recoded gene may be inserted in CP patients, and the mutated copy of the gene might be exchanged using genome editing tools, such as CRISPR/Cas9 [59]. The codon usage studies, therefore, help combat many pancreatitis-associated disorders. 

## 5. Conclusions

Periodontitis is a complex chronic inflammatory disorder caused by various factors acting simultaneously. Most of them are related to the immune fitness of the host. Further studies of genetic and epigenetic alterations in DNA might affect the genetic blueprint of the host immune response. Our study imparts partial knowledge of molecular signatures associated with the set of genes significantly associated with CP. Further studies are warranted to identify more genetic signatures associated with the disease.

## 6. Future Directions and Clinical Implications

CP is a chronic disorder and has shown an association with poor glycemic index, atherosclerosis, hair loss, liver inflammation, arthritis, respiratory problems, anxiety, and other mood disorders. Therefore, knowledge of codon usage and other molecular features in genes responsible for CP may help in gene augmentation through gene therapy with a recoded gene sequence so that gene expression levels may be modulated and brought to a normal level and CP-associated pathophysiology may be alleviated.

## Figures and Tables

**Table 1:** RSCU of NIN, ABHD12B, WHAMM, AP3B3, and SIGLEC6 genes.

Codon	Amino Acid	*NIN*	*ABHD12B*	*WHAMM*	*AP3B3*	*SIGLEC6*
TTT	F	1.395	1.524	1.267	0.571	0.566
TTC		0.605	0.476	0.733	1.429	1.434
TTA	L	0.790	0.816	0.859	0.068	0.114
TTG		0.895	0.893	1.284	0.239	0.321
CTT		1.019	1.106	0.936	0.329	0.712
CTC		0.872	0.553	0.779	0.977	1.594
CTA		0.468	0.512	0.709	0.318	0.093
CTG		1.956	2.120	1.432	4.070	3.167
ATT	I	1.194	1.669	1.258	1.296	0.335
ATC		0.786	1.038	0.842	1.348	2.248
ATA		1.021	0.293	0.901	0.356	0.418
GTT	V	0.806	1.425	1.120	0.390	0.553
GTC		0.851	1.069	0.808	0.827	1.364
GTA		0.704	0.315	0.438	0.422	0.298
GTG		1.639	1.190	1.634	2.361	1.786
TCT	S	1.584	1.861	1.722	0.835	0.646
TCC		1.068	0.233	1.179	1.508	2.378
TCA		0.800	0.297	1.013	0.684	0.402
TCG		0.076	0.630	0.260	0.316	0.629
AGT		1.133	1.796	0.881	1.171	0.090
AGC		1.339	1.184	0.944	1.486	1.856
CCT	P	1.043	0.873	1.083	1.183	0.807
CCC		0.979	1.660	0.645	1.827	1.816
CCA		1.534	1.335	1.542	0.872	1.035
CCG		0.443	0.132	0.729	0.118	0.343
ACT	T	1.071	0.749	1.130	0.597	0.176
ACC		0.990	0.783	1.209	2.507	2.299
ACA		1.447	2.153	1.593	0.726	0.854
ACG		0.491	0.315	0.068	0.171	0.672
GCT	A	1.076	0.902	1.695	0.923	1.236
GCC		1.060	1.280	0.986	1.984	2.031
GCA		1.347	1.634	0.534	0.540	0.599
GCG		0.517	0.184	0.785	0.554	0.135
TAT	Y	1.247	1.449	1.250	0.370	0.720
TAC		0.753	0.551	0.750	1.630	1.280
CAT	H	1.143	0.632	0.794	0.444	0.628
CAC		0.857	1.368	1.206	1.556	1.372
CAA	Q	0.657	0.429	0.749	0.381	0.658
CAG		1.343	1.571	1.251	1.619	1.342
AAT	N	1.140	0.927	1.087	0.617	0.555
AAC		0.860	1.073	0.913	1.383	1.445
AAA	K	1.023	0.803	1.356	0.543	0.549
AAG		0.977	1.197	0.644	1.457	1.451
GAT	D	1.086	1.042	1.229	0.702	0.862
GAC		0.914	0.958	0.771	1.298	1.139
GAA	E	1.072	1.180	1.273	0.424	0.309
GAG		0.928	0.820	0.727	1.576	1.691
TGT	C	1.021	0.973	1.196	1.464	0.687
TGC		0.979	1.027	0.804	0.536	1.313
CGT	R	0.594	1.005	0.560	0.829	0.196
CGC		0.344	0.479	0.691	1.249	1.321
CGA		0.628	0.123	0.501	0.715	0.534
CGG		0.929	0.882	1.088	1.706	0.677
AGA		1.752	2.353	2.009	0.447	1.805
AGG		1.753	1.157	1.151	1.053	1.467
GGT	G	0.968	0.628	1.034	0.406	0.542
GGC		0.988	0.946	1.547	2.069	1.178
GGA		1.036	1.135	0.770	0.698	0.935
GGG		1.009	1.291	0.649	0.828	1.346

**Table 2:** Correlation analysis between overall GC composition and GC composition at all the three codon positions along with CAI and ENc.

	CAI	ENC	%GC	%GC1	%GC2	%GC3
CAI		***	***	NS	*	***
ENC	*−0.945*		***	NS	*	***
%GC	0.808	*−0.819*		NS	***	***
%GC1	*−0.028*	*−0.038*	0.328		NS	NS
%GC2	0.414	*−0.463*	0.667	*−0.134*		*
%GC3	0.897	*−0.864*	0.916	0.278	0.362	

Upper diagonal shows the *p*-value, and the lower diagonal shows the Pearson correlation coefficient. The negative correlation coefficient is shown in italics font, while the positive correlation co-efficient is in straight. Level of significance *** *p* < 0.001; * *p* < 0.05.

**Table 3:** Correlation analysis of PC1 and PC2 with compositional constraints, protein properties (GRAVY and AROMA), and gene expression.

**Component**	**%GC(3)**	**CAI**	**GRAVY**	**AROMA**	**%A**	**%C**	**%T**	**%G**
PC1 (r value)	0.974	0.929	0.345	0.131	*−0.688*	0.853	*−0.577*	0.515
Significance	***	***	NS	NS	***	***	***	***
PC2 (r value)	0.117	0.236	0.755	0.638	*−0.470*	0.002	0.474	0.479
Significance	NS	NS	***	***	**	NS	*	*
**Component**	**%A3**	**%C3**	**%T3**	**%G3**	**%G + C**	**%G3 + C3**	**%A + T**	**%A3 + T3**
PC1 (r value)	*−0.902*	0.887	*−0.837*	0.644	0.881	0.974	*−0.881*	*−0.974*
Significance	***	***	***	***	***	***	***	***
PC2 (r value)	*−0.404*	0.260	0.281	*−0.192*	0.200	0.117	*−0.200*	*−0.117*
Significance	NS	NS	NS	NS	NS	NS	NS	NS

Italics font showed negative correlation coefficient, while straight showed positive correlation coefficient; *** *p* < 0.001; ** *p* < 0.01; * *p* < 0.05; NS: non-significant.

## Data Availability

Available upon request.
